# Novel machine learning models for the prediction of acute respiratory distress syndrome after liver transplantation

**DOI:** 10.3389/frai.2025.1548131

**Published:** 2025-05-27

**Authors:** Weijie Wu, Zheng Zhang, Shuailei Wang, Ru Xin, Dong Yang, Weifeng Yao, Ziqing Hei, Chaojin Chen, Gangjian Luo

**Affiliations:** ^1^Department of Anesthesiology, The Third Affiliated Hospital of Sun Yat-Sen University, Guangzhou, China; ^2^Department of Anesthesiology, The Seventh Affiliated Hospital of Sun Yat-Sen University, Shenzhen, China; ^3^Guangzhou AID Cloud Technology Co., LTD, Guangzhou, China

**Keywords:** liver transplantation, acute respiratory distress syndrome, machine learning, prediction model, random forest

## Abstract

Early prediction of acute respiratory distress syndrome (ARDS) after liver transplantation (LT) facilitates timely intervention. We aimed to develop a predictor of post-LT ARDS using machine learning (ML) methods. Data from 755 patients in the internal validation set and 115 patients in the external validation set were retrospectively reviewed, covering demographics, etiology, medical history, laboratory results, and perioperative data. According to the area under the receiver operating characteristic curve (AUROC), accuracy, specificity, sensitivity, and F1-value, the prediction performance of seven ML models, including logistic regression (LR), decision tree, random forest (RF), gradient boosting decision tree (GBDT), naïve bayes (NB), light gradient boosting machine (LGBM) and extreme gradient boosting (XGB) were evaluated and compared with acute lung injury prediction scores (LIPS). 234 (30.99%) ARDS patients were diagnosed. The RF model had the best performance, with an AUROC of 0.766 (accuracy: 0.722, sensitivity: 0.617) in the internal validation set and a comparable AUROC of 0.844 (accuracy: 0.809, sensitivity: 0.750) in the external validation set. The performance of all ML models was better than LIPS (AUROC 0.692, 0.776). The predictor variables included the age of the recipient, BMI, MELD score, total bilirubin, prothrombin time, operation time, standard urine volume, total intake volume, and red blood cell infusion volume. We firstly developed a risk predictor of post-LT ARDS based on RF model to ameliorate clinical practice.

## 1 Introduction

For patients with end-stage liver disease, liver transplantation (LT) is currently the most effective treatment (Samuel et al., 2024), while acute respiratory distress syndrome (ARDS) is a common postoperative complication following liver transplantation (LT) with high morbidity and mortality rates. In recent studies, ARDS after LT has been reported to cause a range of morbidities, with an incidence between 1 and 30% (Feltracco et al., 2013). ARDS plays a pivotal role in the poor survival of post-LT patients, leading to prolonged intensive care unit (ICU) and hospital stays, increased in-hospital mortality, and long-term physical, psychological, and social disabilities (Gorman et al., 2022; Oh et al., 2023).

Although postoperative ARDS significantly impacts the clinical outcomes of LT patients, it is often unrecognized and underdiagnosed, leading to the underutilization of timely and effective treatments (Tariparast et al., 2025). Therefore, the current research efforts mainly focus on identifying the risk factors which assist clinicians in implementing preventive interventions in the early stage (Grasselli et al., 2023). Earlier studies have reported many risk factors related to ARDS after LT, and these risk factors include recipient age, smoking history, ongoing dialysis, and preoperative total bilirubin (Ripoll et al., 2020). In addition, relevant prediction models, such as acute lung injury (ALI) prediction scores (LIPS), were established to predict ARDS/ALI (Kim B. K. et al., 2021). Using routinely available clinical data, we can identify patients at high risk of ARDS/ALI by LIPS in the early stage of their illness (Wei et al., 2025). However, the prediction performance of LIPS in post-LT patients has not been reported. Furthermore, the nonlinear relationship between the outcome variables and the explanatory variables could not be excluded, and the overfitting and multicollinearity limitations could not be avoided during traditional regression analysis (Bayman and Dexter, 2021).

Novel applications of machine learning (ML) methods in medicine have emerged and are constantly evolving (Gotlieb et al., 2022). ML has the ability to minimize the above limitations of regression analysis and to analyze large, complex datasets, yielding sophisticated outcomes and prediction models (Rubulotta et al., 2024). ML methods have already been applied in different fields in transplant medicine (Bhat et al., 2023), including organ allocation, prediction of overall survival, and short-and long-term complications, resulting in the development of significant prediction models with the potential to improve clinical practice (Swinckels et al., 2024; Tran et al., 2022). However, currently, no studies have reported the performance of machine learning models in predicting postoperative ARDS in LT patients (Tran et al., 2024).

Therefore, we tried to use our perioperative database to determine risk factors for perioperative ARDS in adult LT patients. Moreover, we compared the prediction performance of LIPS and seven machine learning models, including logistic regression (LR), random forest (RF), decision tree (DT), gradient boosting decision tree (GBDT), naïve bayes (NB), light gradient boosting machine (LGBM), and extreme gradient boosting (XGB). Finally, a visualized risk predictor based on an optimal machine learning model was developed to predict post-LT ARDS at ICU admission.

## 2 Methods

### 2.1 Study design and subjects

This was a retrospective study conducted at a single center and was approved by the Ethics Committee of the Third Affiliated Hospital of Sun Yat-sen University ([2021]02-023-01). We retrospectively reviewed the electronic medical records of 952 LT patients in our institution between January 2015 and February 2020. The recipients of organ transplantation were all registered in the China Organ Transplant Response Systems (www.cot.org.cn). During retrospective enrollment, patients who were under the age of 18 (*n* = 111), underwent combined organ transplantation (*n* = 13), presented with preoperative ARDS (*n* = 24) or were missing sufficient data (*n* = 49) were excluded from this study. A total of 755 patients were included in the final cohort, which was used to develop and internally validate the prediction models of postoperative ARDS in LT patients.

Meanwhile, according to the same inclusion and exclusion criteria, 143 patients who underwent LT from March 2020 to December 2020 in our institution were screened for temporal external validation of the prediction models. Among the 143 identified patients, those who were under the age of 18 (*n* = 21), underwent combined organ transplantation (*n* = 1), presented with preoperative ARDS (*n* = 2) or were missing sufficient data (*n* = 4) were excluded. A total of 115 patients were included in the external validation set.

As the primary outcome of our analysis, ARDS was identified according to the Berlin Definition (Ranieri et al., 2012), including PaO2/FiO2 ≤ 300 mmHg within 7 days after liver transplantation, respiratory failure not fully explained by fluid overload or cardiac failure, and bilateral opacities consistent with pulmonary edema on a chest radiograph. The secondary outcomes included lengths of stay (ICU, hospital), overall hospitalization cost, and one-year survival of patients after liver transplantation.

**Figure 1:**
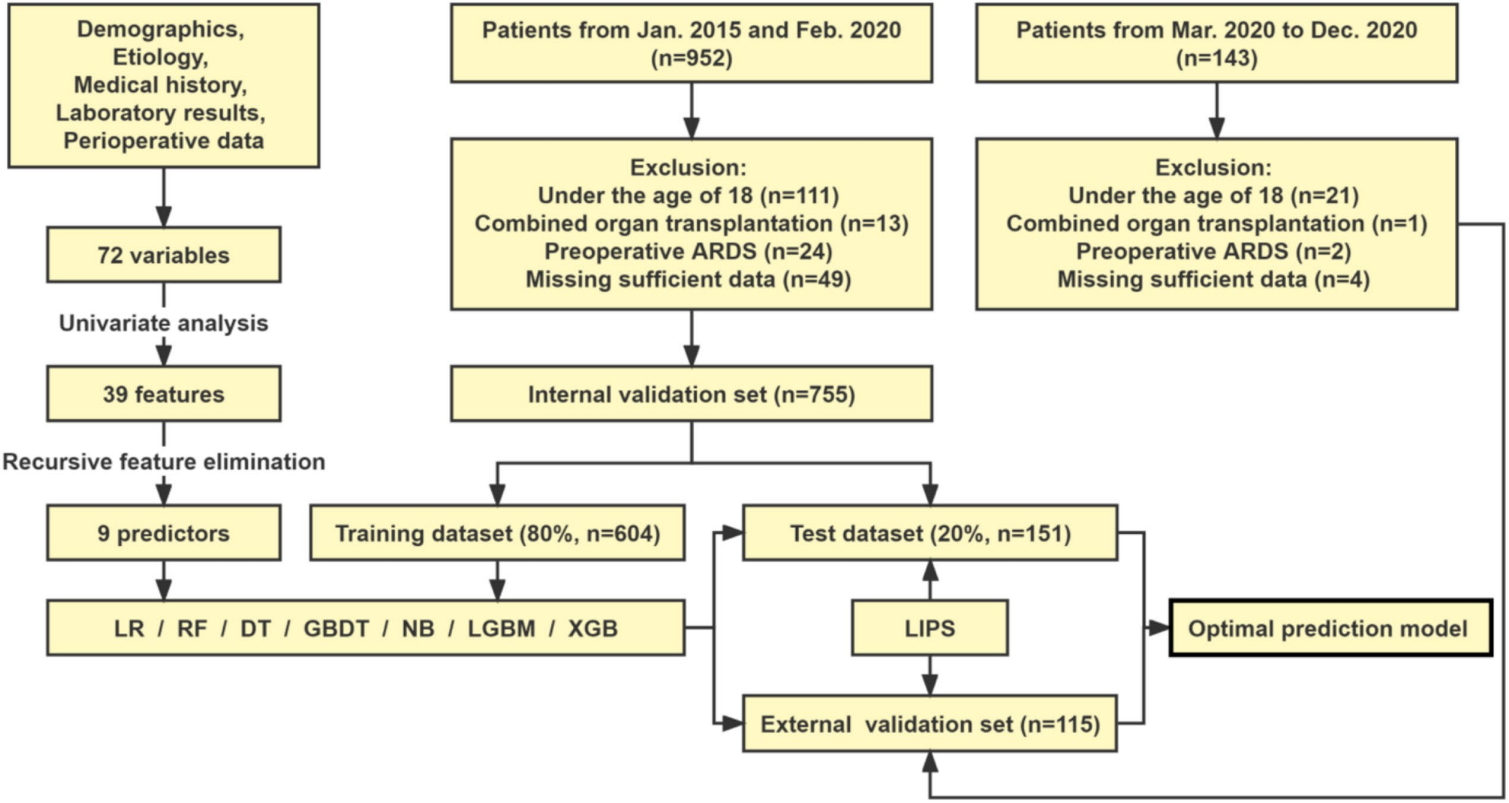
Study flowchart. LR, logistic regression; DT, decision tree; RF, random forest; GBDT, gradient boosting decision tree; NB, naive bayes; LGBM, light gradient boosting machine; XGB, extreme gradient boosting.

In addition, the predictive capability of the models was compared with LIPS, which is currently used to predict the ARDS/ALI risk. Finally, the optimal prediction model was visualized as an online risk predictor for clinical application at ICU admission. The flow diagram of this study is presented in Figure 1.

### 2.2 Data collection

In the EPR systems of our institution, a database platform was established by extracting medical records from the hospital information system, picture archiving, and communication system, laboratory information system, and care anesthesia system. The medical data chosen for our analysis were extracted from this database platform in our hospital and were grouped into the following categories: (1) Demographics: age of recipient, gender and body mass index (BMI); (2) Etiology for liver transplantation: hepatitis, hepatocellular carcinoma, alcoholic liver cirrhosis, acute hepatic failure, cholestatic liver cirrhosis, genetic metabolic diseases and other reasons; (3) Comorbidities: hypertension, diabetes mellitus, cardiovascular disease, chronic kidney disease, cerebrovascular disease, bronchiectasis, old tuberculosis, chronic obstructive pulmonary disease (COPD), pulmonary hypertension, lung infection, pulmonary nodules, smoking history and alcohol history; (4) Complications and treatments: use of preoperative continuous renal replacement therapy (CRRT), plasma exchange (PE) and respirator, hepato-pulmonary syndrome, hepato-renal syndrome, pleural effusion, spontaneous bacterial peritonitis, hepatic encephalopathy, thrombogenesis, esophageal and gastric varices, model for end-stage liver disease (MELD) score and Child–Turcotte–Pugh score; (5) Laboratory results: white blood cells, red blood cells, platelet, hemoglobin, aspartate transaminase, alanine transaminase, albumin, total bilirubin, concentration of K^+^, Na^+^, Ca^2+^, Cl^−^, HCO_3_^−^ in blood, blood glucose, creatinine, urea nitrogen, fibrinogen, prothrombin time and international normalized ratio; (6) Surgery and anesthesia characteristics: operation time, anesthesia time, cold ischemic time and anhepatic phase time; (7) Intraoperative fluid administration and transfusions: crystalloid, colloid, sodium bicarbonate, albumin, urine output, standardize urinary output, blood loss, ascites removal, the total amount, the total output, red blood cell transfusion, fresh frozen plasma transfusion, cryoprecipitate transfusion and platelet transfusion. A total of 72 potential perioperative predictive variables were included in the initial analysis.

### 2.3 Variable selection

**Figure 2:**
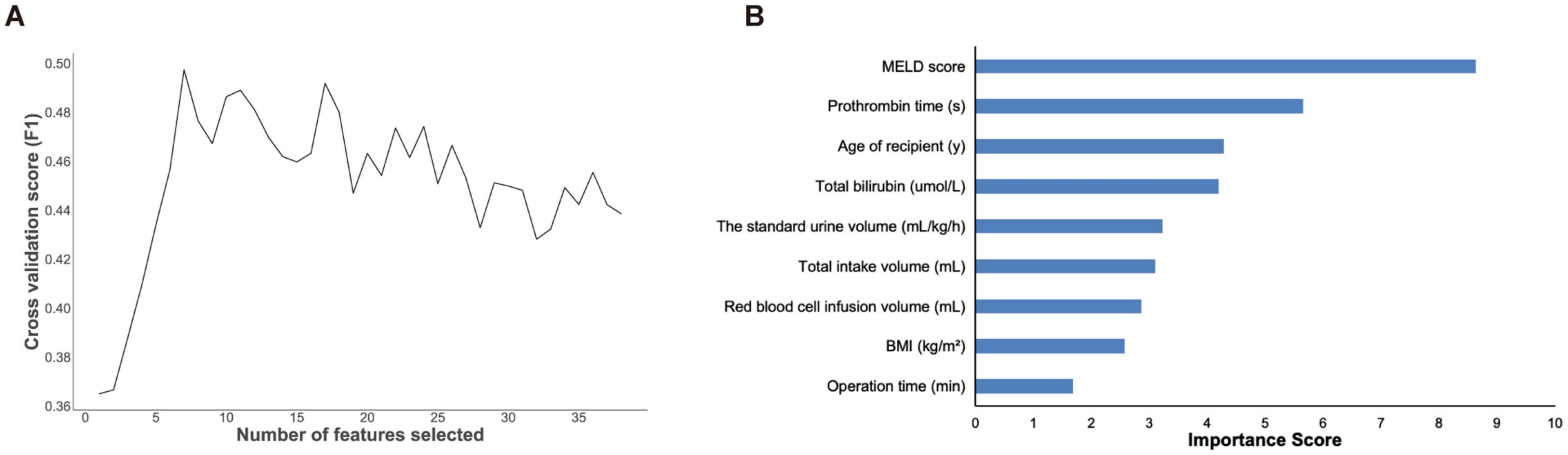
The results of features screening and selection. **(A)** Number of features screened by RFE method. **(B)** Feature importance ranking of the selected nine features illustrated by random forest. MELD, model for end-stage liver Disease; BMI, body mass index.

To reduce the effects of overfitting during training for the model performance, we implemented a univariate analysis to filter out the 39 statistically significant features among the 72 variables. The statistically significant features in the univariate analysis were screened by 5-fold cross-validation (Liu et al., 2019) and the recursive feature elimination (RFE) method embedded with random forest (Li et al., 2024), which was trained on the above variables. The least important variables were then recursively removed. We finally selected the subset of variables with the highest F1-value to develop machine learning prediction models. As shown in Figure 2, the results showed that the model performed best when the number of features was nine. Therefore, the nine features included the age of the recipient, BMI, MELD score, total bilirubin, prothrombin time, operation time, standard urine volume, total intake volume, and red blood cell infusion volume. In addition, the rank of feature importance was determined.

### 2.4 Classification algorithms

To systematically evaluate the predictive capacity of machine learning (ML) in post-LT ARDS, we implemented seven algorithms representing diverse computational paradigms. These models were selected based on their established performance in medical data analysis, methodological heterogeneity, and ability to address clinical challenges such as imbalanced outcomes and feature interactions (Haug and Drazen, 2023; Liu et al., 2019). These models were developed using Scikit-learn, XGBoost, and LightGBM libraries with hyperparameters optimized through 5-fold cross-validation. Missing values were addressed via median/mode imputation consistent with clinical data preprocessing standards. The comparative optimization algorithm is outlined below.

(1) Logistic Regression (LR): A linear probabilistic model serving as the baseline for its interpretability and compatibility with traditional clinical risk scoring systems. LR provides odds ratios that align with conventional statistical analyses while accommodating nonlinear relationships via feature engineering (Zabor et al., 2022). (2) Decision Tree (DT): A rule-based classifier generating transparent decision pathways through recursive binary splitting. DT was included to establish interpretable decision thresholds and contrast performance against ensemble methods (Luo et al., 2022). (3) Random Forest (RF): An ensemble of 100 decor-related decision trees utilizing bootstrap aggregation and randomized feature subset selection. Chosen for its intrinsic overfitting resistance and ability to model complex interactions between surgical parameters and biochemical markers through feature importance ranking (Becker et al., 2023). (4) Gradient Boosting Decision Tree (GBDT): A sequential boosting algorithm optimizing prediction residuals through additive tree construction. Selected for its superior performance on imbalanced datasets via adaptive instance reweighting (Seto et al., 2022), critical given the 30.99% ARDS incidence rate. (5) Naïve Bayes (NB): A probabilistic classifier assuming conditional feature independence. Implemented for computational efficiency in real-time clinical settings and tolerance to minor data missingness (Zhang, 2016). (6) Light Gradient Boosting Machine (LGBM): High-performance gradient boosting framework employing histogram-based optimization and leaf-wise tree growth. Adopted to efficiently process temporal intraoperative variables while minimizing computational overhead (Yanagawa et al., 2024). (7) eXtreme Gradient Boosting (XGB): Regularized gradient boosting with sparsity-aware split finding and L2 penalty terms. Utilized to handle heterogeneous clinical data types while maintaining model generalizability through strict regularization constraints (Hou et al., 2020).

### 2.5 Model training and evaluation

The patients were randomly divided into a training dataset (80%) and a test dataset (20%). Patients from the training dataset were used to develop machine learning models. Patients from the testing dataset were used to validate and compare the performance of the developed-models. We used 5-fold cross-validation to determine the optimal hyperparameter combination of each machine learning method. The hyperparameters with the highest average validation area under the receiver operating characteristic curve (AUROC) were considered the optimal hyperparameters. Furthermore, we used 500 bootstrap resamples to calculate the 95% confidence intervals around the sample correlation estimates. Missing data were present in less than 5% of the total records. Moreover, we substituted the mean for the missing data for continuous variables and the mode for incidence variables.

The prediction capability of the machine learning models was assessed and compared according to AUROC, accuracy, specificity, sensitivity, and F1-value. Accuracy indicates the percent of correct prediction among all the samples. Specificity illustrates the correct prediction ratios of the negative samples, while sensitivity denotes the correct prediction ratios of the positive samples. The F1-value is a comprehensive evaluation index that combines precision and sensitivity.

### 2.6 Statistical analysis

Our analysis was performed using the Python programming language (Python Software Foundation, version 3.7.4). We expressed continuous variables as the medians (interquartile ranges) and categorical variables as numbers (percentages). Continuous variables were analyzed using the nonparametric Mann–Whitney U test and Wilcoxon W-test. Categorical variables were compared using the chi-squared test with continuity correction. Statistical significance was determined by a *p*-value less than 0.05.

## 3 Results

### 3.1 The basic information and prognosis of the study subjects

A total of 755 patients were included in the final cohort and were used to develop and internally validate machine learning models for predicting postoperative ARDS. It is noteworthy that ARDS occurred in 234 patients after liver transplantation, accounting for 30.99% of the study subjects, while 521 (69.01%) patients did not have ARDS. The basic clinical characteristics of the enrolled patients are presented in Table 1.

**Table 1:** Clinical basic characteristics.

Variables	Patients without ARDS	Patients with ARDS	*p*-value
	(*n* = 521)	(*n* = 234)	
Demographics			
Age of recipient (years)	48 (14.0)	50 (16.0)	0.011
Gender (female)	74 (14.2%)	27 (11.5%)	0.379
BMI (kg/m^2^)	22 (4.1)	23 (4.5)	0.001
Etiology for liver transplantation			
Hepatitis (*n*)	118 (22.7%)	69 (29.5%)	0.055
Hepatocellular carcinoma (*n*)	243 (46.6%)	53 (22.7%)	<0.001
Alcoholic liver cirrhosis (*n*)	14 (2.7%)	9 (3.9%)	0.530
Acute hepatic failure (*n*)	118 (22.7%)	96 (41.0%)	<0.001
Cholestatic liver cirrhosis (*n*)	20 (3.8%)	2 (0.9%)	0.043
Genetic metabolic diseases (*n*)	7 (1.3%)	5 (2.1%)	0.623
Other reasons (*n*)	1 (0.2%)	0 (0.0%)	0.681
Comorbidities			
Hypertension (*n*)	47 (9.0%)	28 (12.0%)	0.263
Diabetes mellitus (*n*)	78 (15.0%)	35 (15.0%)	0.916
Cardiovascular disease (*n*)	17 (3.3%)	15 (6.4%)	0.073
Chronic kidney disease (*n*)	6 (1.2%)	3 (1.3%)	0.834
Cerebrovascular disease (*n*)	4 (0.8%)	2 (0.9%)	0.750
Bronchiectasis (*n*)	12 (2.3%)	2 (0.9%)	0.283
Old tuberculosis (*n*)	16 (3.1%)	5 (2.1%)	0.629
COPD (*n*)	15 (2.9%)	13 (5.6%)	0.111
Pulmonary hypertension (*n*)	2 (0.4%)	2 (0.9%)	0.778
Lung infection (*n*)	343 (65.8%)	190 (81.2%)	<0.001
Pulmonary nodules (*n*)	170 (32.6%)	47 (20.1%)	0.001
Smoking history (*n*)	162 (31.1%)	101 (43.2%)	0.002
Alcohol history (*n*)	134 (25.7%)	84 (35.9%)	0.006
Complications and treatments			
Use of preoperative CRRT (*n*)	25 (4.8%)	35 (15.0%)	<0.001
Use of preoperative PE (*n*)	23 (4.4%)	28 (12.0%)	<0.001
Use of preoperative respirator (*n*)	15 (2.9%)	32 (13.7%)	<0.001
Hepato-pulmonary syndrome (*n*)	0 (0.0%)	0 (0.0%)	1.000
Hepato-renal syndrome (*n*)	8 (1.5%)	22 (9.4%)	<0.001
Pleural effusion (*n*)	139 (26.7%)	74 (31.6%)	0.191
Spontaneous bacterial peritonitis (*n*)	46 (8.8%)	39 (16.7%)	0.002
Hepatic encephalopathy (*n*)	87 (16.7%)	85 (36.3%)	<0.001
Thrombogenesis (*n*)	30 (5.8%)	12 (5.1%)	0.859
Esophageal and gastric varices (*n*)	327 (62.8%)	126 (53.9%)	0.026
MELD score	17 (19.0)	33 (18.8)	<0.001
Child–Turcotte–Pugh score	9 (5.0)	11 (3.0)	<0.001
Laboratory results			
White blood cells (10^9^/L)	5.0 (4.3)	6.7 (6.6)	<0.001
Red blood cells (10^12^/L)	3.3 (1.6)	3.0 (1.4)	0.001
Platelet (10^9^/L)	75.0 (87.0)	67.5 (66.8)	0.023
Hemoglobin (g/L)	102.0 (40.0)	96.0 (37.0)	0.022
Aspartate transaminase (U/L)	60.0 (75.0)	81.0 (85.5)	<0.001
Alanine transaminase (U/L)	38.0 (44.0)	49.0 (78.3)	0.003
Albumin (g/dL)	35.6 (7.2)	35.2 (5.9)	0.253
Total bilirubin (umol/L)	72.2 (380.3)	363.6 (449.2)	<0.001
K^+^ (mmol/L)	3.8 (0.6)	3.9 (0.6)	0.918
Na^+^ (mmol/L)	139.5 (6.4)	138.8 (6.1)	0.129
Ca^2+^ (mmol/L)	2.3 (0.2)	2.3 (0.3)	0.006
Cl^−^ (mmol/L)	103.1 (8.5)	101.2 (8.9)	0.074
HCO_3_^−^ (mmol/L)	22.8 (4.2)	22.4 (4.9)	0.655
Blood glucose (mmol/L)	4.9 (2.0)	5.0 (3.3)	0.730
Creatinine (umol/L)	72.0 (27.0)	72.5 (46.5)	0.105
Urea nitrogen (mmol/L)	3.8 (3.3)	2.9 (3.3)	<0.001
Fibrinogen (g/L)	2.7 (2.9)	2.0 (3.6)	0.043
Prothrombin time (s)	18.2 (13.2)	31.0 (19.8)	<0.001
International normalized ratio	1.6 (1.4)	3.0 (2.3)	<0.001
Surgery and anesthesia characteristics			
Operation time (min)	420 (100.0)	440 (100.8)	<0.001
Anesthesia time (min)	505 (110.0)	527 (112.0)	<0.001
Cold ischemic time (min)	360 (77.0)	360 (75.0)	0.223
Anhepatic Phase time (min)	45 (13.0)	47 (15.0)	0.057
Intraoperative fluid and transfusion			
Crystalloid (mL)	3,000 (1500.0)	3,200 (1600.0)	0.269
Colloid (mL)	0 (0.0)	0 (0.0)	0.447
Sodium bicarbonate (mL)	0 (125.0)	110 (250.0)	<0.001
Albumin (mL)	250 (150.0)	250 (200.0)	0.043
Urine output (mL)	1,600 (1200.0)	1,200 (1385.0)	<0.001
Standardize urinary output (mL/kg/h)	3.1 (2.2)	2.4 (2.1)	<0.001
Blood loss (mL)	1,000 (1200.0)	1948 (2000.0)	<0.001
Ascites removal (mL)	300 (1500.0)	1,000 (1800.0)	<0.001
The total amount (mL)	7,090 (3190.0)	8,450 (4343.8)	<0.001
The total output (mL)	3,650 (2800.0)	4,280 (3880.0)	<0.001
Red blood cell transfusion (mL)	1,000 (1070.0)	1,500 (1500.0)	<0.001
Fresh frozen plasma transfusion (mL)	2,400 (1200.0)	3,000 (1662.5)	<0.001
Cryoprecipitate transfusion (unit)	30 (20.0)	36 (10.5)	<0.001
Platelet transfusion (*n*)	38 (7.3%)	33 (14.1%)	0.005

Data were presented as median (interquartile range) or number (%).

The patients with ARDS had significantly increased ICU stay hours (169.1 vs. 79.3 h, *p* < 0.001), hospital stay days (26.9 vs. 24.5 d, *p* = 0.003), total hospitalization cost (413513.0 vs. 305144.8, p < 0.001), and decreased 7-day survival rate (94.0% vs. 98.7%, *p* = 0.001), 1-month survival rate (82.9% vs. 96.0%, *p* < 0.001), 6-month survival rate (76.5% vs. 92.7%, *p* < 0.001), 1-year survival rate (73.5% vs. 88.7%, *p* ≤ 0.001). The postoperative prognosis of the LT patients is shown in Table 2.

**Table 2:** The postoperative prognosis of LT patients.

Variables	Patients without ARDS	Patients with ARDS	*p*-value
ICU stay hours	79.3 (74.4)	169.1 (130.0)	<0.001
Hospital stay days	24.5 (13.7)	26.9 (15.5)	0.003
Total hospitalization cost	305144.8 (109557.5)	413513.0 (161721.3)	<0.001
7-days survival	514 (98.7%)	220 (94.0%)	0.001
1-month survival	500 (96.0%)	194 (82.9%)	<0.001
6-months survival	483 (92.7%)	179 (76.5%)	<0.001
1-year survival	462 (88.7%)	172 (73.5%)	<0.001

Data were presented as median (interquartile range) or number (%).

### 3.2 Internal validation performance of the machine learning models

**Figure 3:**
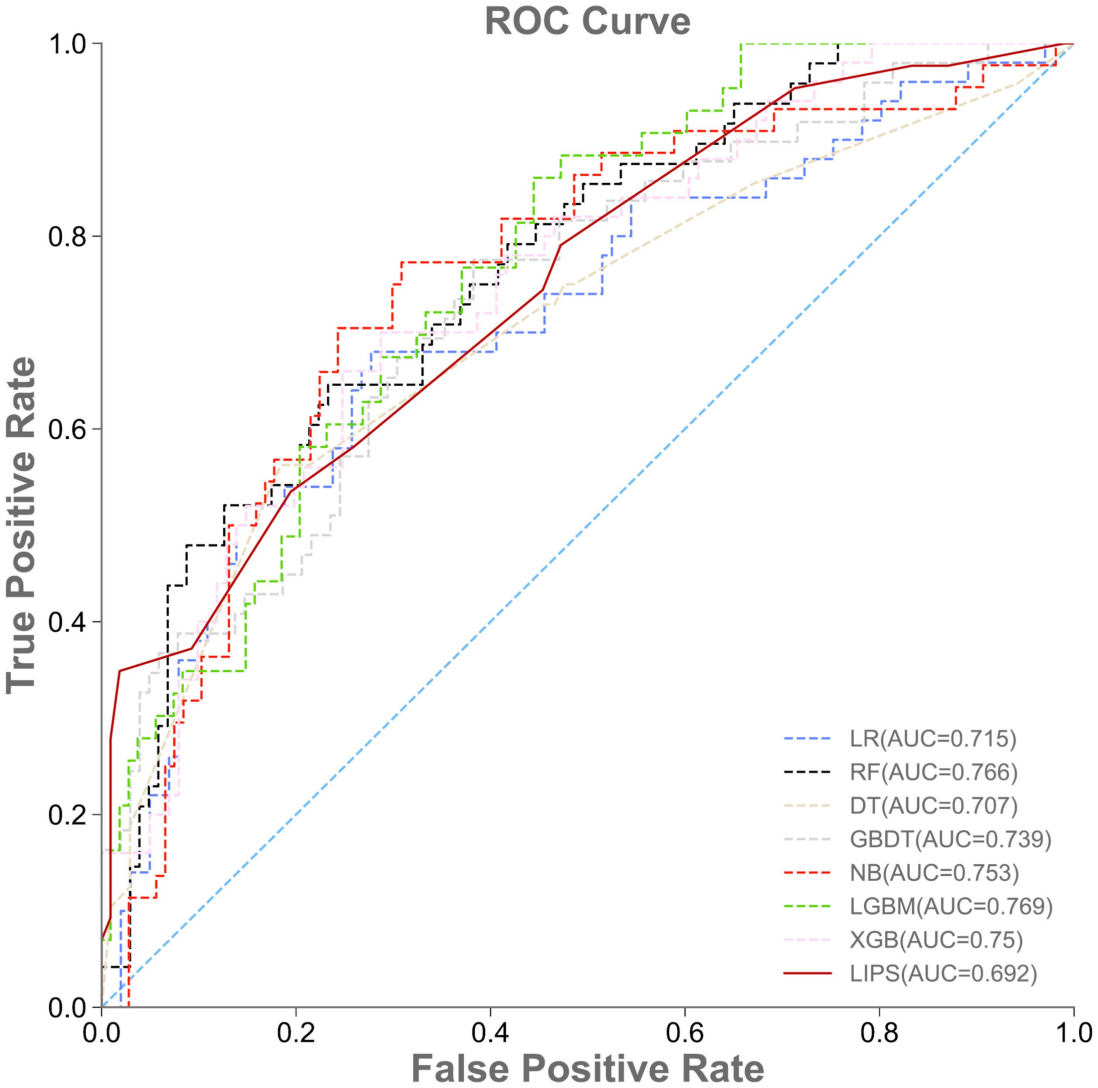
Performance of machine learning models and LIPS in the internal validation set. ROC, area under the receiver operating characteristic curve.

The AUROC, accuracy, specificity, sensitivity, and F1-value of the internal validation of the machine learning models are shown in Table 3 and Figure 3. Among the seven models, LGBM had the largest AUROC (0.769, 95% CI 0.699–0.830) and highest specificity (0.700, 95% CI 0.350–1.000). DT had the smallest AUROC (0.707, 95% CI 0.617–0.781). RF (0.766, 95% CI 0.693–0.825) had a better AUROC than the other models, with the exception of LGBM. XGB showed the highest accuracy (0.735, 95% CI 0.665–0.795), while LR had the lowest accuracy (0.649, 95% CI 0.579–0.715) and the highest sensitivity (0.682, 95% CI 0.548–0.814). RF had the highest F1-value (0.574, 95% CI 0.467–0.667). Since the RF model had the greatest comprehensive prediction performance in the internal validation set, we eventually chose the RF model for further analysis and application.

**Table 3:** Performance of machine learning models and LIPS in the internal validation set.

Models	AUROC	Accuracy	Specificity	Sensitivity	F1-value
LR	0.715 (0.632–0.787)	0.649 (0.579–0.715)	0.458 (0.353–0.558)	0.682 (0.548–0.814)	0.547 (0.442–0.637)
RF	0.766 (0.693–0.825)	0.722 (0.652–0.781)	0.540 (0.422–0.661)	0.617 (0.488–0.735)	0.574 (0.467–0.667)
DT	0.707 (0.617–0.781)	0.675 (0.589–0.755)	0.481 (0.362–0.611)	0.641 (0.449–0.8)	0.551 (0.429–0.64)
GBDT	0.739 (0.662–0.803)	0.728 (0.662–0.792)	0.600 (0.433–0.777)	0.391 (0.265–0.537)	0.470 (0.351–0.585)
NB	0.753 (0.676–0.819)	0.728 (0.656–0.788)	0.565 (0.419–0.716)	0.520 (0.394–0.659)	0.545 (0.412–0.643)
LGBM	0.769 (0.699–0.830)	0.728 (0.649–0.801)	0.700 (0.350–1.000)	0.245 (0.019–0.440)	0.359 (0.038–0.53)
XGB	0.750 (0.678–0.811)	0.735 (0.665–0.795)	0.606 (0.457–0.761)	0.423 (0.291–0.554)	0.494 (0.378–0.596)
LIPS	0.692 (0.601–0.774)	0.722 (0.695–0.748)	0.608 (0.500–0.696)	0.370 (0.300–0.462)	0.459 (0.390–0.507)

Data were presented as mean (95% confidence interval).

### 3.3 External validation performance of the machine learning models

**Figure 4:**
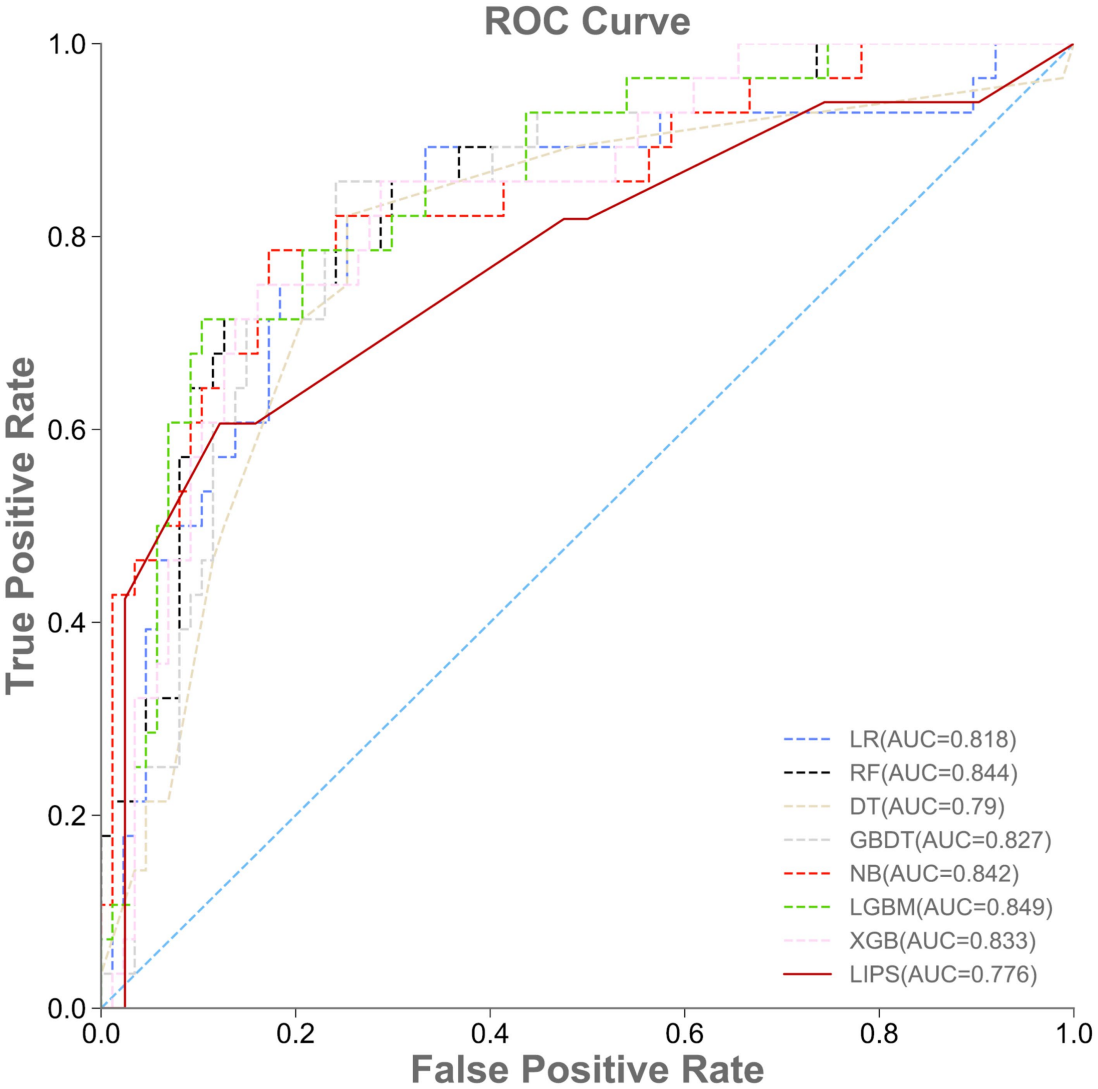
Performance of machine learning models and LIPS in the external validation set. ROC, area under the receiver operating characteristic curve.

A total of 115 patients were included in the external validation set, including 28 (24.35%) with ARDS and 87 (75.65%) without ARDS. The AUROC, accuracy, specificity, sensitivity, and F1-value of the various machine learning models are shown in Table 4 and Figure 4. Among the seven models, LGBM had the largest AUROC (0.849, 95% CI 0.831–0.865) and highest specificity (0.667, 95% CI 0.158–1.000). DT had the smallest AUROC (0.790, 95% CI 0.661–0.855). Similarly, the AUROC of the RF model (0.844, 95% CI 0.823–0.862) was better than that of the other models but worse than that of the LGBM model. NB showed the highest accuracy (0.826, 95% CI 0.817–0.835), while LR showed the lowest accuracy (0.748, 95% CI 0.713–0.783) and the highest sensitivity rate (0.821, 95% CI 0.750–0.857). RF had the highest F1-value (0.646, 95% CI 0.606–0.688). The RF model also had the best comprehensive performance for predicting ARDS after LT in the temporal validation set.

**Table 4:** Performance of machine learning models and LIPS in the external validation set.

Models	AUROC	Accuracy	Specificity	Sensitivity	F1-value
LR	0.818 (0.799–0.833)	0.748 (0.713–0.783)	0.489 (0.451–0.535)	0.821 (0.75–0.857)	0.611 (0.568–0.658)
RF	0.844 (0.823–0.862)	0.809 (0.783–0.826)	0.583 (0.538–0.625)	0.750 (0.679–0.786)	0.646 (0.606–0.688)
DT	0.790 (0.661–0.855)	0.765 (0.678–0.826)	0.514 (0.400–0.617)	0.714 (0.536–0.857)	0.597 (0.476–0.696)
GBDT	0.827 (0.795–0.860)	0.809 (0.769–0.843)	0.630 (0.532–0.727)	0.536 (0.393–0.643)	0.577 (0.468–0.667)
NB	0.842 (0.831–0.851)	0.826 (0.817–0.835)	0.654 (0.630–0.680)	0.607 (0.571–0.643)	0.630 (0.611–0.655)
LGBM	0.849 (0.831–0.865)	0.791 (0.757–0.835)	0.667 (0.158–1.000)	0.304 (0.017–0.571)	0.421 (0.031–0.627)
XGB	0.833 (0.805–0.860)	0.809 (0.765–0.843)	0.609 (0.520–0.692)	0.571 (0.464–0.679)	0.593 (0.500–0.673)
LIPS	0.776 (0.657–0.880)	0.809 (0.783–0.843)	0.688 (0.588–0.786)	0.458 (0.387–0.519)	0.531 (0.468–0.593)

Data were presented as mean (95% confidence interval).

### 3.4 Comparison of the prediction performance: the machine learning models vs. LIPS

The prediction performance of the lung injury prediction score (LIPS) in the internal validation set is shown in Table 3 and Figure 3. The AUROC was 0.692, 95% CI 0.601–0.774. The accuracy was 0.722, 95% CI 0.695–0.748. The specificity was 0.608, 95% CI 0.500–0.696; the sensitivity was 0.370, 95% CI 0.300–0.462; and the F1-value was 0.459, 95% CI 0.390–0.507. The prediction performance of the LIPS model in the temporal validation set is shown in Table 4 and Figure 4: the AUROC was 0.776, 95% CI 0.657–0.880; the accuracy was 0.809, 95% CI 0.783–0.843; the specificity was 0.688, 95% CI 0.588–0.786; the sensitivity was 0.458, 95% CI 0.387–0.519; and the F1-value was 0.531, 95% CI 0.468–0.593. Surprisingly, the prediction performance of all the machine learning models was better than that of LIPS. These results indicated the poor ability of the LIPS model to predict postoperative ARDS in LT patients.

### 3.5 The SHAP value and feature importance

**Figure 5:**
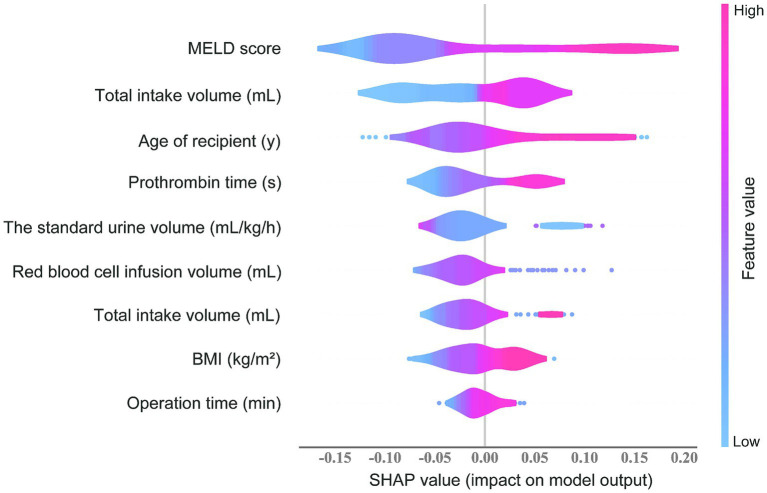
The SHAP value plot by combining feature importance with feature effects in RF model.

The feature importance evaluated using the SHAP value in the RF prediction model is shown in Figure 5. MELD score, prothrombin time, and red blood cell infusion volume ranked as the top three important predictors. The transparency of the prediction made by the RF model was increased according to the SHAP summary plot. Each point represents a sample, and a wide area means a large number of samples are gathered. The color on the right indicates the value of the feature, red indicates that the feature value is high, and blue indicates that the feature value is low. Therefore, the results showed that the age of the recipient, BMI, MELD score, total bilirubin, prothrombin time, operation time, total intake volume, and red blood cell infusion volume was associated with higher SHAP value output, indicating a higher likelihood of ARDS after liver transplantation. Conversely, the standard urine volume was associated with a lower probability of postoperative ARDS. These results are consistent with what we observed in clinical practice.

To achieve clinical application and improve clinical practice, we developed a forecasting website based on the RF model. There were nine blank inset boxes for users to input the main relevant variables, and the incidence of ARDS after LT could be calculated and shown on this page. The results were expressed as a binary outcome with the probability (%) of developing postoperative ARDS. Visit the website at http://wb.aidcloud.cn/zssy/ards.html.

## 4 Discussion

Early recognition and prompt medical intervention of postoperative ARDS in LT patients are imperative for diminishing the risks of ARDS progression and improving the survival rate of patients (Wu et al., 2022). A reliable and accurate prediction model is needed to improve the prognosis of ARDS after LT. In our study, for the early prediction of postoperative ARDS after LT, we developed and validated the prediction performance of seven machine learning models and LIPS. The results showed that the random forest model performed optimally in predicting ARDS after LT among the above prediction models. Moreover, the RF model indicated that the age of the recipient, BMI, MELD score, total bilirubin, prothrombin time, operation time, standard urine volume, total intake volume, and red blood cell infusion volume were the nine most important weights for ARDS after LT. Based on the RF model, we developed an online risk predictor for timely detection and intervention at ICU admission, and this predictor can ultimately improve clinical practices.

In the last few decades, the current donor allocation system prioritized patients with the most severe liver disease (Lee et al., 2019), as evidenced by a large percentage of patients with multiorgan dysfunction and high MELD scores in our study cohort (Kim W. R. et al., 2021). Therefore, various underlying respiratory disorders might be present in patients with end-stage liver disease before transplantation. Our study showed that the length of ICU stay, postoperative hospital stay, hospitalization cost, and mortality rate in LT patients suffering from ARDS were increased significantly. The findings of the present study were consistent with those of previous studies (Ripoll et al., 2020). Notably, our results demonstrated that the incidence of ARDS was 30.99%. However, the studies available in the literature with a comparable LT population reported an incidence of ARDS of less than 5% after the introduction of the Berlin definition (Ripoll et al., 2020; Zhao et al., 2016). One probable reason was that the proportion of LT patients with severe hepatitis and acute liver failure in our center was significantly higher than the proportion of patients in the above two studies. Higher degrees of hepatic impairment resulted in a higher incidence of postoperative ARDS (Ketcham et al., 2020).

To date, ML prediction models have already shown excellent performance in predicting diseases and clinical conditions, and personalized risk probabilities could be generated for each patient (Abdullah, 2024; Maddali et al., 2022). These models could provide decision-support tools to assist clinicians in targeting interventions (Cheng et al., 2025). After the development and validation of various machine learning models, our study showed that the RF model had the best performance in both internal (AUROC: 0.766) and external validation (AUROC: 0.844) for predicting ARDS after LT. This can be attributed to RF’s ensemble structure, which aggregates predictions from multiple decision trees to minimize overfitting, and its ability to handle nonlinear relationships through feature importance ranking. In contrast, simpler models like LR and DT showed lower sensitivity and specificity, likely due to their linear assumptions or susceptibility to noise. LGBM achieved the highest AUROC in external validation (0.849) but exhibited lower sensitivity (0.304), suggesting a trade-off between specificity and sensitivity in imbalanced datasets. Random forest is an extension of the traditional decision tree classifier. Each tree was constructed from a random subset of the explanatory variables and a random subset of the original training data. By voting for these randomly generated trees, random forests could minimize overfitting by making the decision (Hu and Szymczak, 2023).

For statistical validation, we divided 500 repeat samples of patients into different training and test datasets (80% training and 20% test sets) and 5-fold cross-validation confirmed the robustness of RF, with tight confidence intervals for AUROC and F1-scores (Tables 3, 4). The results of the training dataset were validated using a temporal external validation set. Remarkably, in our study, some of the data distributions of the internal and temporal external validation sets had significant differences, reflecting the strong robustness and adaptability of the RF model on data with different distributions. Moreover, the wide application of structured data and database systems has created the technical foundation for applying complex big data algorithms in clinical settings (Swinckels et al., 2024). In this study, the perioperative database system included a variety of in-hospital data such as demographic data, medical history, preoperative test and examination results, and anesthetic and surgical records, as shown in Tables 1, 2.

Previous studies have shown that a variety of risk factors are associated with postoperative ARDS (Ketcham et al., 2020; Rubulotta et al., 2024). All features of the RF model identified in our study were in accordance with previous research on the risk factors for ARDS, and these results reflect the advantage of capturing correlations between independent variables in large complex datasets and finding trends in subsets of data. Our study highlights the limitations of the LIPS model, which showed suboptimal performance in predicting post-LT ARDS (AUROC: 0.692–0.776). Unlike LIPS, which relies on linear risk scoring, ML models like RF dynamically integrate nonlinear interactions between variables (e.g., the interplay between MELD score and red blood cell transfusions). This aligns with recent literature emphasizing the superiority of ML in complex clinical scenarios. For instance, the SHAP analysis of the RF model revealed that intraoperative factors (e.g., red blood cell infusion volume) and preoperative liver dysfunction (e.g., MELD score) synergistically contribute to ARDS risk—a relationship undetectable by traditional scoring systems. These findings underscore the need for data-driven, ML-based tools in perioperative critical care. In addition, the features are routinely recorded and widely used in clinical practice, and none of these factors are obtained by special instruments or equipment, indicating that our model is feasible and suitable for use in hospitals in a wide range of settings. Although the internal mechanisms remain unclear, the high clinical relevance of these factors has established a solid foundation for the subsequent development of machine learning models and has made the conclusions clinically and practically valuable.

Finally, we developed an internet-based risk estimator based on the RF model of this study, which was easy to use for clinicians. The forecasting website could facilitate the translation and clinical application of our research findings (Li et al., 2022). Our model was able to calculate the likelihood of developing postoperative ARDS in LT patients at ICU admission, allowing the output to reflect the risk of the target event rather than just providing a binary outcome. A definite time window before the event would probably make potential intervention more realistic. However, to optimize the model performance and to improve the accuracy of risk prediction, prospective multicenter datasets should be collected to validate the prediction performance of our machine learning model in the future.

This study had several limitations. First, this study is a single-center retrospective study with a small sample size. The results need to be interpreted with caution because they lacked variables of intraoperative respiratory parameters, which might be critical. Second, it is more difficult to interpret the results of machine learning models than traditional methods. The different datasets in machine learning models might show different performances and results. Additionally, AUROC values could vary depending on the different parameters used in machine learning. The models need to correspond to the different occasions based on the requirements. Excessive prioritization of AUC values should be avoided because this might make models unreliable in real-world applications. Third, many of the important variables reported in our study are not clinically modifiable. Therefore, it is not certain that our results could turn into a viable alternative to improve the clinical outcomes of patients undergoing liver transplantation. However, personalized prevention might be appropriate based on risk information.

## 5 Conclusion

In conclusion, this study focused on ARDS, a life-threatening complication following liver transplantation, by constructing machine learning predictive models through the integration of multidimensional perioperative clinical data. Feature selection identified nine core predictors. Comparative analysis revealed that predictive models developed using seven machine learning algorithms demonstrated significantly superior performance to conventional pulmonary injury prediction scores, with the random forest model exhibiting optimal predictive capability. To facilitate clinical translation, we developed an online risk calculator (accessible at http://wb.aidcloud.cn/zssy/ards.html) based on the optimal model, which enables real-time individualized risk assessment through a dynamic interactive interface, thereby providing decision support for preoperative risk evaluation and intraoperative precision management. The findings not only enhance understanding of risk factors for post-transplant ARDS but more importantly establish a clinically applicable intelligent early-warning system, demonstrating substantial practical implications for improving patient prognosis.

## Data Availability

The raw data supporting the conclusions of this article will be made available by the authors, without undue reservation.
